# Anatomical organization of MCH connections with the pallidum and dorsal striatum in the rat

**DOI:** 10.3389/fnsys.2014.00185

**Published:** 2014-10-01

**Authors:** Sandrine Chometton, Vesna Cvetkovic-Lopes, Christophe Houdayer, Gabrielle Franchi, Amandine Mariot, Fabrice Poncet, Dominique Fellmann, Pierre-Yves Risold

**Affiliations:** EA3922, SFR FED 4234, UFR Sciences Médicales et Pharmaceutiques, Université de Franche-ComtéBesançon, France

**Keywords:** neuroanatomy, basal nuclei, subthalamic nucleus, lateral hypothalamus, arousal

## Abstract

Neurons producing the melanin-concentrating hormone (MCH) are distributed in the posterior hypothalamus, but project massively throughout the forebrain. Many aspects regarding the anatomical organization of these projections are still obscure. The present study has two goals: first to characterize the topographical organization of neurons projecting into the cholinergic basal forebrain (globus pallidus, medial septal complex), and second to verify if MCH neurons may indirectly influence the dorsal striatum (caudoputamen) by innervating afferent sources to this structure. In the first series of experiments, the retrograde tracer fluorogold was injected into multiple sites in the pallidal and medial septal regions and the distribution of retrogradely labeled neurons were analyzed in the posterior lateral hypothalamus. In the second series of experiments, fluorogold was injected into the caudoputamen, and the innervation by MCH axons of retrogradely labeled cells was analyzed. Our results revealed that the MCH system is able to interact with the basal nuclei in several different ways. First, MCH neurons provide topographic inputs to the globus pallidus, medial septal complex, and substantia innominata. Second, striatal projecting neurons in the cortex, thalamus, and substantia nigra presumably receive only sparse inputs from MCH neurons. Third, the subthalamic nucleus is heavily innervated by MCH projections, thus, presumably serves as one important intermediate station to mediate MCH influence on other parts of the basal nuclei.

## Introduction

Melanin-concentrating hormone (MCH)-containing neurons in the hypothalamus send extensive projections throughout the cerebral cortex (Bittencourt et al., 1992; Risold et al., 1997). In addition, MCH neurons also abundantly innervate several subcortical structures in the basal forebrain that are connected with the cortex. For example, MCH axons innervate cholinergic neurons in the medial septal and diagonal band nuclei and influence memory formation in the hippocampus through this pathway (Chung et al., 2011; Lu et al., 2013). A role for MCH in the motivation of feeding is pointed out in the nucleus accumbens which expresses high levels of the MCH-R1 receptor (Georgescu et al., 2005; Pissios et al., 2008; Guesdon et al., 2009; Sears et al., 2010; Hopf et al., 2013). The nucleus accumbens is heavily connected with the prefrontal cortical areas, ventral pallidum, amygdalar nuclei, and ventral tegmental area. MCH cortical projections also involve the whole isocortex. To date, the general organization of the circuitry that link the MCH system to the isocortical and classical extrapyramidal circuitries is still little investigated. We can found in the literature evidence of MCH projections in the globus pallidus, and more than 10 years ago interactions between the MCH system and the mesostriatal pathway was suspected (Knigge et al., 1996). However, the overall organization of an anatomical circuitry linking the MCH system to the striato-pallido-nigral pathway is unknown at this point.

The aims of the present study was dual: first, to analyze with some details in the rat the topographical organization of MCH neurons projecting into the whole cholinergic basal telencephalon (globus pallidus, substantia innominata and medial septal complex) and second, to identify possible pathways that MCH neurons may use to indirectly influence isocortical output through the dorsal striatum. To achieve our two goals, we performed retrograde tracer injections through the pallidal ventral forebrain of rats and analyzed the expression of MCH in retrogradely labeled cells in the lateral hypothalamic area (LHA). We also made injections in the dorsal striatum and verified whether MCH axons innervate those retrogradely labeled neurons in the other parts of the brain, including the cerebral cortex, thalamus, subthalamus, and substantia nigra.

## Materials and methods

### Animals

Sprague–Dawley male rats, weighing 280–380 g, were obtained from Janvier (Le Genest-Saint-Isle, France). They were housed under 12 h light: 12 h dark cycle at a constant room temperature and had free access to water and standard laboratory diet. All animal use and care protocols were in accordance with institutional guidelines (all protocols were approved and investigators authorized).

### Tracer injections

Rats were anesthetized with an intramuscular (IM) injection of a mixture of xylazine and ketamine (Vetokinol, 1 mg/100 g of body weight and 10 mg/100 g, respectively) and placed in a stereotaxic instrument.

Rats received a unilateral iontophoretic injection of 10% Fluorogold (FG, Interchim) solution diluted in 0.9% NaCl. Coordinates were taken according to the Paxinos and Watson stereotaxic atlas (Paxinos and Watson, 2005). Injections were made in different sites of the basal telencephalon (medial septal complex, nucleus of the diagonal band, substantia innominata, globus pallidus, and dorsal striatum). Glass micropipettes (tip diameter: 30–50 μm) were used to inject the FG iontophoretically into these regions using an intermittent current of 5 μA and 7 s on/off time for 5 min. The micropipette was left in place for another 5 min before being removed to avoid FG diffusion along the micropipette track.

After 10 days survival time, rats were deeply anesthetized with intraperitoneal injection (IP) of Pentobarbital (CEVA, 50 mg/kg). Animals were perfused transcardially with 0.9% NaCl followed by ice-cold 4% paraformaldehyde (PFA, Roth) fixative in 0.1 M phosphate buffer saline (PBS) at pH 7.4. Brains were removed, post-fixed in the same fixative for several hours at 4°C, immersed overnight at 4°C in a solution of 15% sucrose in 0.1 M PBS, and then quickly frozen. Brains were cut in four series of 30 μm coronal thick sections, collected in a cryoprotector solution (1:1:2 glycerol/ethylene glycol/PBS), and stored at −40°C.

### Immunohistochemistry FG

After rinsing in PBS + 0.3% Triton X100, free-floating sections were incubated with the primary anti-FG antiserum raised in rabbit (polyclonal, Oncogene Research Products) at a dilution of 1:3000 in PBS containing 0.3% Triton X100, 1% bovine serum albumin, 10% lactoproteins, and 0.01% sodium azide, during 65 h at 4°C. Then, free-floating sections were incubated for 24 h at 4°C in a solution of biotinylated goat anti-rabbit IgG antibody (Vector Laboratories) at a dilution of 1:1000 in PBS Triton. Finally, sections were placed in the mixed avidin-biotin horseradish peroxidase (HRP) complex solution (ABC Elite Kit, Vector Laboratories) for 1 h at room temperature. The peroxidase complex was visualized by an exposure to a chromogen solution containing 0.04% 3,3′diaminobenzidine tetrahydrochloride (DAB, Sigma) and 0.006% hydrogen peroxide (Sigma) in PBS at pH 7.4. The reaction was stopped by extensive washing in PBS at pH 7.4. Free-floating sections were mounted on gelatin-coated slides, and then dehydrated and coverslipped with Canada balsam (Roth). An adjacent series was always stained in a solution of 1% toluidine blue (Roth) in water to serve as a reference series for cytoarchitectonic purposes.

### Triple staining FG/MCH/CART

Sections were incubated with the anti-MCH antibody (rabbit polyclonal, our laboratory, Risold et al., 1992) dissolved in PBS containing 0.3% Triton X100, 1% bovine serum albumin, 10% lactoproteins, and 0.01% sodium azide at 1:1000 for 65 h at 4°C. Tissues were then incubated with Alexa Fluor 488 goat anti-rabbit IgG antibody (Invitrogen) diluted in PBS-T at 1:1000 for 2 h at room temperature.

After the first staining, sections were incubated with the anti-CART antibody (Cocaine and Amphetamine Regulated Transcript, mouse monoclonal, generously provided by Dr J. T. Clausen, Novo Nordisk, Denmark, 1:1000) dissolved in PBS-T for 65 h at 4°C. Tissues were then incubated with Alexa Fluor 555 donkey anti-mouse IgG antibody (Invitrogen) diluted in PBS-T at 1:1000 for 2 h at room temperature. Finally, free-floating sections were mounted on gelatin-coated slides and coverslipped with 60:40 glycerol:PBS-T.

The auto-fluorescence of FG was observed under UV illumination.

### Double staining MCH/parvalbumin or MCH/ChAT

The MCH is revealed by the technique described above. Then, sections were incubated with the anti-parvalbumin (mouse monoclonal, Swant) or anti-ChAT (goat polyclonal, AB144P Chemicon) antibodies dissolved in PBS containing 0.3% Triton X100, 1% bovine serum albumin, 10% lactoproteins and 0.01% sodium azide at 1:1000 for 65 h at 4°C. Tissues were then incubated with Alexa Fluor 555 donkey anti-mouse IgG antibody (Invitrogen) or Cyanine-3 donkey anti-goat IgG antibody (Jackson Immunoresearch) diluted in PBS-T at 1:1000 for 2 h at room temperature. Finally, free-floating sections were mounted on gelatin-coated slides and coverslipped with 60:40 glycerol:PBS-T.

## Results

### Topographical organization of MCH projections in the cholinergic basal telencephalon

In this work, we mostly employed the nomenclature of Swanson (2004). The magnocellular cholinergic neurons in the basal forebrain are abundant across the borders of the medial septal complex (medial septal nucleus and nucleus of the diagonal band), the magnocellular preoptic nucleus, the substantia innominata and most the internal segment of the globus pallidus. However, in the globus pallidus and the medial septal complex, these cholinergic neurons are segregated within a medial sector (peripheral in the diagonal band nucleus), while an external part (central in the diagonal band nucleus) is rich in parvalbumin-containing neurons (Kiss et al., 1990; Hontanilla et al., 1998; Henderson et al., 2004; Risold, 2004; Croizier et al., 2010; Mallet et al., 2012). Such differentiation of parvalbumin positive vs. cholinergic-rich region is not as clear in the substantia innominata and in the magnocellular preoptic nucleus.

#### Distribution of MCH axons in the cholinergic-rich pallidal basal telencephalon

**Figure 1:**
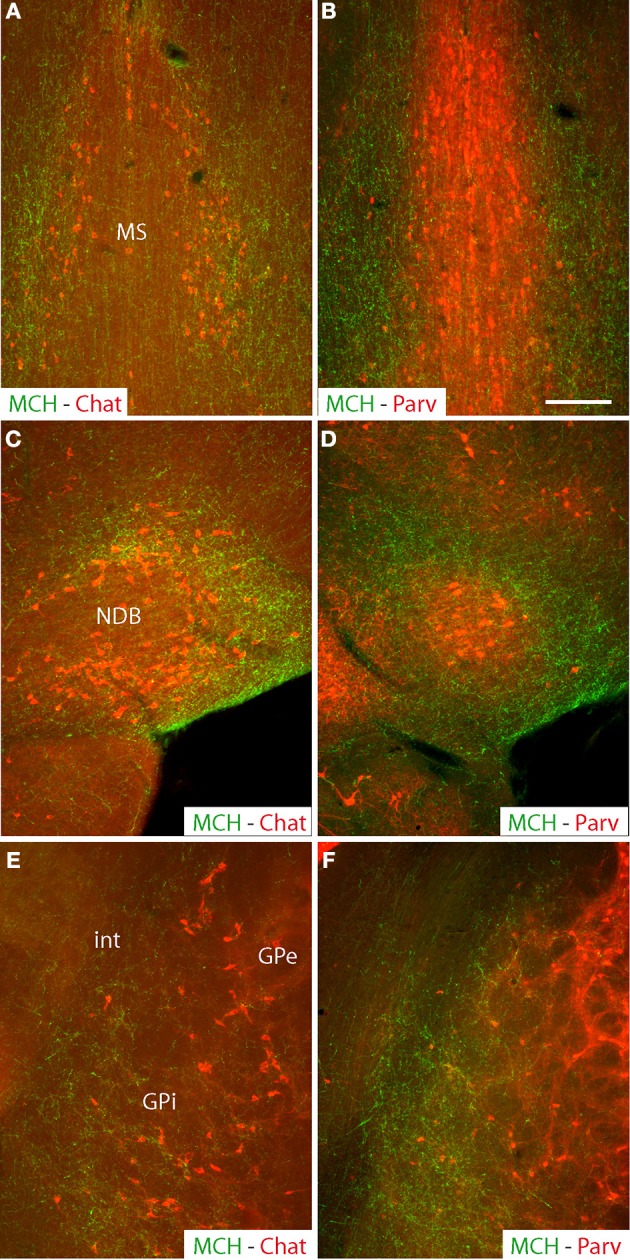
**Photomicrographs of coronal sections labeled by double immunohistochemistry to illustrate the distribution of MCH axons (green) in comparison with distributions of cholinergic neurons labeled by an anti-Chat antibody (red), or parvalbumin-expressing neurons (red) in the medial septal nucleus (A,B) the nucleus of the diagonal band (C,D) or the globus pallidus (E,F)**. Note that in all of these three structures, MCH axons are more abundant in cholinergic-rich parts, but relatively sparse in the parvalbumin-rich parts. Scale bar = 100 μm.

MCH projections arising from the medial forebrain bundle are abundant in the cholinergic rich regions of both the *medial septal complex and the globus pallidus*, while the parvalbumin-rich part of the septum received “en passant” inputs as already reported elsewhere (Croizier et al., 2010) (Figure 1). This “en passant” input is far less intense in the external segment of the globus pallidus.

Within the *substantia innominata*, MCH axons are especially abundant in the posterior part, compared to the rostral regions and the magnocellular preoptic nucleus that were only diffusely innervated.

Images evocating a direct innervation by MCH axons of cholinergic neurons was observed in all nuclei of the basal forebrain, confirming already recently published information (Lima et al., 2013) and is not further illustrated in the present work. Numerous buttons were observed close to non-ChAT positive cells, clearly suggesting that MCH axons innervate other cell populations than the cholinergic neurons in these structures. As previously reported (Cvetkovic et al., 2004), most of these axons expressed both MCH and CART. MCH/non-CART axons were obviously less numerous.

#### Origin of the hypothalamic projections into the cholinergic basal telencephalon

**Figure 2:**
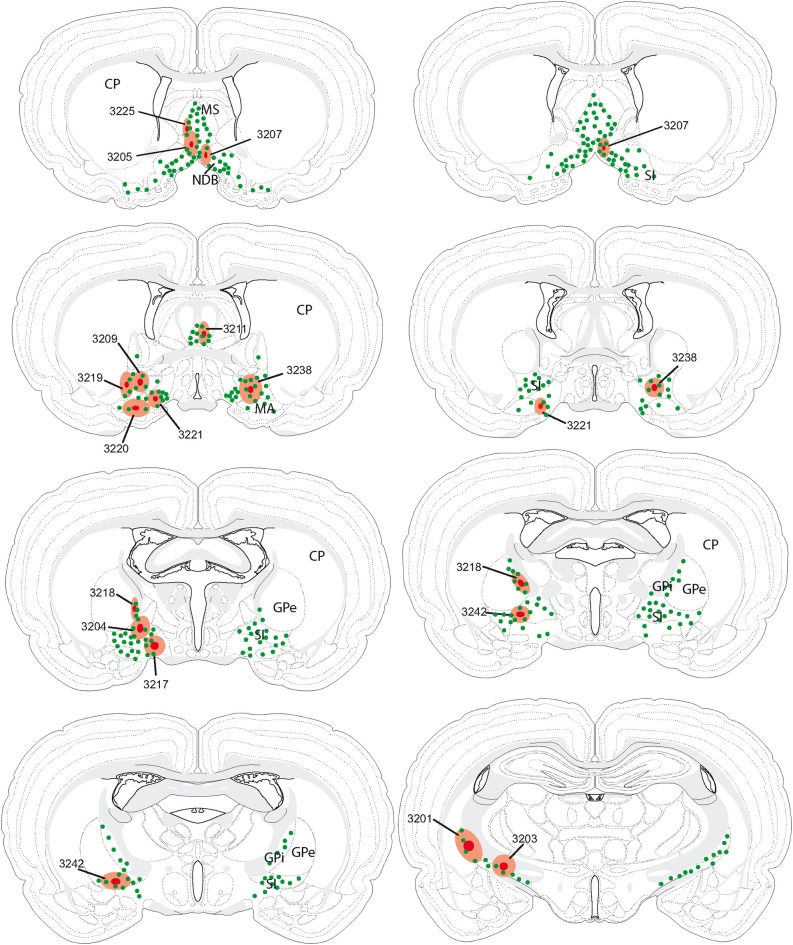
**Illustration of all fluorogold injection sites in comparison with the distribution of cholinergic neurons in the basal telencephalon (green dots)**. In several experiments (individually identified by a four digit number), injection sites were centered (dark red spot) in the medial septal nucleus, nucleus of the diagonal band, substantia innominata or internal segment of the globus pallidus. The hallo around this center (light red area) may slightly extend beyond the borders of targeted nuclei (see also Table 1).

**Figure 3:**
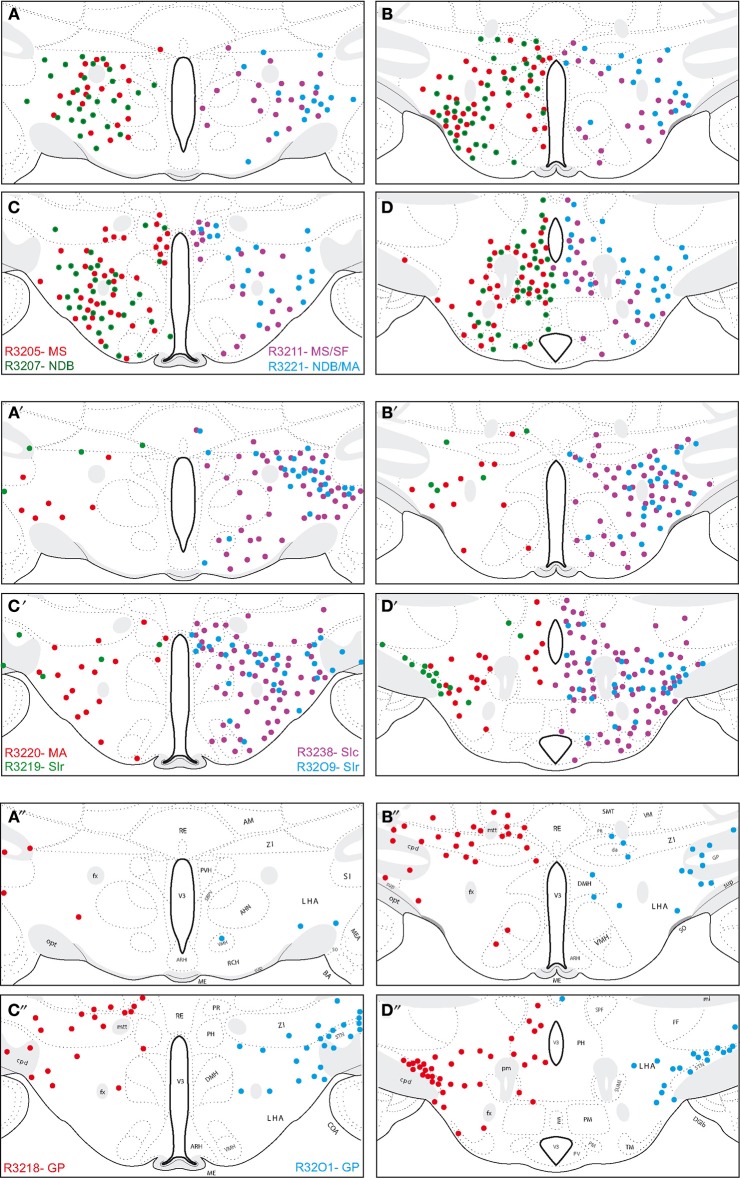
**Schematic distribution of neurons retrogradely labeled by the fluorogold after injections in the basal telencephalon (Figure 2 and Table 1 for injection sites)**. The distribution of these neurons is mapped on four sections in the coronal planes through the posterior hypothalamus. They are organized from rostral **(A)** to caudal **(D)**. Four (upper two panels; 2 on the left and 2 on the right) or two (lower panel) experiments are illustrated per panels. Note the topographical organization of retrogradely labeled cells depending on the location of the injection sites (see text for details).

The retrograde tracer fluorogold (FG) was injected into the cholinergic basal forebrain. Several injection sites were restricted to the medial septal nucleus, diagonal band nucleus, substantia innominata or internal segment of the globus pallidus (Table 1). Their extent through the rostrocaudal basal telencephalon is schematized in Figure 2. The distribution of retrogradely labeled cells in the LHA is schematized in Figure 3. Retrogradely labeled neurons within the LHA were abundant when injection sites involved the medial septal complex or caudal parts of the substantia innominata, but the number of retrogradely labeled cells was low when injections sites were centered in the magnocellular preoptic nucleus, rostral substantia innominata or globus pallidus.

**Table 1:** **Injection sites in the Pallidum**.

**Rats no**.	**FG Injection sites**
R3205	Ventral MS/dorsal NDB
R3225	Lateral edge of the MS
R3207	Dorsal NDB
R3211	Caudal MS/septofimbrial nucleus
R3221	NDB/MA
R3209	SIr
R3219	SIr
R3220	MA
R3204	SIc
R3242	SIc
R3238	SIc
R3218	Internal segment of the GP
R3201	Caudal internal segment of the GP

A clear topographical organization could be recognized with retrogradely labeled neurons being more medial when the injection sites were centered into the medial septal complex. In such cases, retrogradely labeled neurons were abundant in the hypothalamic medial zone. In the LHA proper, the ventral perifornical parts contained the densest condensations of retrogradely labeled neurons. Parts of the medial zona incerta also contained some cells. When injection sites were centered in the caudal substantia innominata, medial zone nuclei as the ventromedial hypothalamic nucleus were labeled, but in the LHA, retrogradely labeled neurons were more laterally observed. When injection sites involved the rostral substantia innominata, very few neurons were found medially in the hypothalamus, only the LHA contained a significant number of FG expressing cells. Finally, after injections in the globus pallidus, retrogradely labeled cells were far less abundant in the hypothalamus, mostly in lateral parts of the LHA close to the cerebral peduncle. The lateral zona incerta contained also some FG expressing cells. By contrast, the adjacent subthalamic nucleus contained a very intense labeling after globus pallidus injections, as expected.

#### Origin of the MCH projections into the cholinergic basal telencephalon

**Figure 4:**
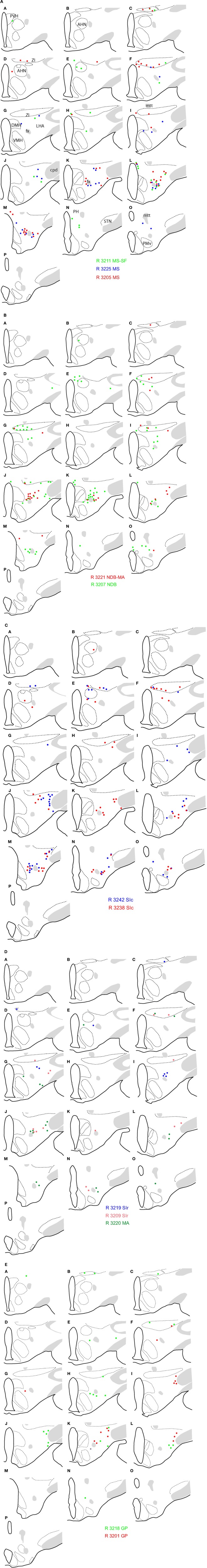
**(A–E)** Distribution of FG-MCH neurons in the posterior hypothalamus after different FG injections in the basal telencephalon. This distribution is mapped on coronal drawings of the caudal hypothalamus arranged from rostral **(a)** to caudal **(p)**. These drawings were produced from Nissl stained sections and mapping was made with regard to the distribution of MCH cell bodies from an adjacent series of section. Therefore, results of all FG-injected brains were represented on this set of reference drawings and with regard to cytoarchitecture and to MCH distribution. One dot on the drawing corresponds to one MCH cell labeled by the fluorogold observed on the section. After septal injections **(A,B)** retrogradely labeled MCH neurons were medial in the hypothalamus, while after injection in the substantia innominata **(C,D)** or globus pallidus **(E)** retrogradely labeled perikarya were more lateral. Note the dense group of retrogradely labeled cells in the posterior perifornical region **(j–m)**.

Using double labeling procedures, many FG-labeled neurons in the hypothalamus proper were also recognized by the MCH-antiserum. The distribution of these cells is reported in the Figure 4. Several point retained our attention:

First, similarly to the whole hypothalamic distribution, medial cells, including in the zona incerta, tended to be labeled by the FG when the injection sites involved the medial septal complex (Figures 4A,B), while after globus pallidus injections mostly MCH neurons close to the cerebral peduncle contained the retrograde labeling (Figure 4E). We did not perform a quantitative analysis as the number of retrogradely labeled cells is clearly dependent of the size of the injection site. Nevertheless, retrogradely labeled MCH neurons appeared to be more abundant when the tracer was injected into the medial septal or posterior regions of the substantia innominata (Figures 4A–C) than into the rostral substantia innominata or globus pallidus (Figures 4D,E). However, MCH-FG neurons after septal or substantia innominata injections represented only a fraction of the whole number of FG retrogradely labeled cells in the hypothalamus. On the contrary, most FG cells in the LHA after the globus pallidus injections were labeled by the MCH-antiserum.
Second, a caudal perifornical MCH condensation, at caudal levels of the dorsomedial hypothalamic nucleus (Figures 4A–D section levels **j–l**), was a major source of MCH projections to the cholinergic medial septal complex, as retrogradely labeled cells were systematically numerous within this condensation in such cases (Figure 4E). By contrast, very caudo-medial MCH cell bodies adjacent and in the posterior periventricular nucleus were never retrogradelly labeled in any of the experiments.
Finally, we already reported in the past that most MCH projections in the telencephalon also contained CART. We also confirmed this trend in the present study and as previously mentioned (Cvetkovic et al., 2004), around 80% of MCH-FG neurons were also labeled by CART antibodies (not shown).

### Indirect MCH projections into the dorsal striatum

MCH projections are sparse within the dorsal striatum. Fibers labeled for MCH traveled essentially through ventral components of the internal capsule. Only the caudal tail of the caudoputamen nucleus contained some axons providing a very moderate to light innervations of the neuropil.

**Figure 5:**
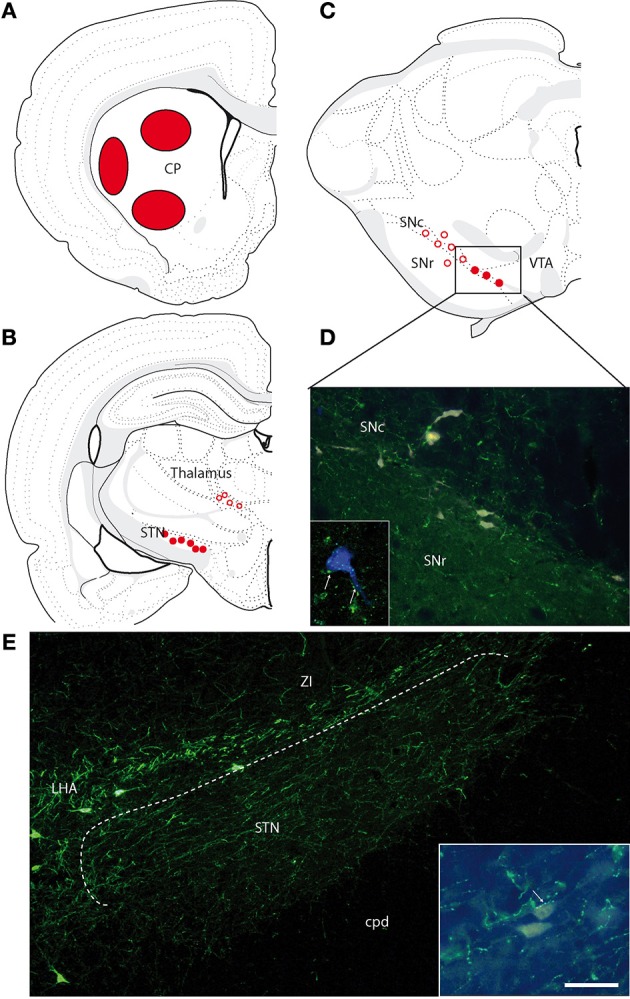
**(A)** Location of the FG injection sites in the caudoputamen nucleus. **(B,C)** Brief illustration of the distribution of retrogradely labeled cells at thalamic **(B)** and midbrain **(C)** levels after FG injection in the caudoputamen nucleus and represented on schematized coronal sections from Swanson (2004). Open circles represent the distribution of retrogradely labeled cells that were very little innervated by MCH axons. Filled dots represent the distribution of retrogradely labeled cells that were contacted by MCH axons. **(D)** Photomicrographs illustrating FG-expressing cells in the ventral substantia nigra compacta. MCH-labeled axons are observed close to some of these neurons. Framed figure is a confocal micrograph illustrating a MCH axons (in green, arrows) contacting a FG expressing cell (in blue to increase contrast). **(E)** Photomicrographs illustrating the MCH innervations of the subthalamic nucleus. Framed figure is a micrograph illustrating a MCH axons (in green, arrows) contacting a FG labeled neurons. Scale bar = 100 μm (30 μm for framed pictures).

Four FG injections were aimed at the dorsal striatum, and three were restricted to respectively, dorsal, ventral, or ventrolateral portions of the caudoputamen nucleus (Figure 5A). Retrogradely labeled cells were observed in the cerebral cortex, the globus pallidus, intralaminar nuclei of the thalamus, the subthalamic nucleus, the dorsal raphe nucleus, and the substantia nigra. Only one or two per experiment were found within the LHA/zona incerta. These very few cells were labeled by the MCH-antiserum (not shown).

MCH labeled axons were examined in proximity of retrogradely labeled cells in the following regions.

#### In the cerebral cortex

Many retrogradely labeled neurons were observed dorsally or more laterally in the layer 5 of the isocortex, depending on the location of the injection sites in the striatum. MCH projections are moderately abundant through the isocortex. Although those MCH axons were often seen in the vicinity of these neurons, very few putative contacts were observed. Therefore, it is unlikely that MCH axons provide significant innervation onto those corticostriatal projecting neurons.

#### In the globus pallidus

Some retrogradely labeled cells were observed in the nucleus. An occasional buttons at immediate proximity of retrogradely labeled cells could be noticed, but most of the retrogradely labeled neurons were not approached by MCH axons.

#### In the dorsal thalamus

Retrogradely labeled cells were observed in the central lateral and parafascicular nuclei. Again, few MCH axons were seen at the immediate vicinity of retrogradely labeled cells.

#### In the subthalamic nucleus

Many FG-labeled neurons are distributed in this nucleus. It contains also moderate to intense innervation by MCH axons. Many images evocating synaptic contacts were observed between MCH-labeled axons and FG-expressing perikarya. Almost every FG-expressing perikarya seemed contacted by one or several MCH buttons (Figures 5B,E). However, fluorogold containing neurons in the subthalamic nucleus were far more abundant after injections in the globus pallidus.

#### In the substantia nigra

This structure contained the brightest FG neurons after caudoputamen injections. As expected, these cells were mostly observed in the substantia nigra pars compacta, but a few were found in the substantia nigra pars reticulata, or in the adjacent ventral tegmental area. In the pars compacta of the substantia nigra, these cells were distributed in the ventromedial or more dorsal regions. Dorsal cells were not innervated by MCH axons. On the contrary, retrogradely labeled cells in the ventromedial pars compacta were obviously targeted by MCH axons (Figures 5C,D).

#### In the dorsal raphe

The dorsal raphe nucleus is known to send a serotonergic input to the dorsal striatum, and some retrogradely labeled cells were observed within this nucleus. MCH axons were seen close to these cells that may receive a moderate MCH innervation.

## Discussion

The basal telencephalon is an essential target of MCH axons in all vertebrates, from agnathians to mammalians (Croizier et al., 2013). In rodents, the literature data implicating MCH in several structures of the basal forebrain, i.e., the nucleus accumbens and the medial septal complex, is now quite abundant. However, interactions between the MCH system and the striatonigral pathways have been little investigated so far. In the present study, we observed several strong anatomical links between the MCH system and the striatonigral pathways:

First, MCH projections are abundant in all structures that contain magnocellular cholinergic neurons, from the medial septal complex to the internal segment of the globus pallidus. The distribution of the neurons of origin is topographically organized in the lateral hypothalamus. These projections may contact cholinergic but also non-cholinergic cells.
Second, besides to the internal segment of the globus pallidus, MCH axons innervate the subthalamic nucleus.

### Organization of MCH projections into the pallidum

Both the globus pallidus and the medial septal complex are divided into parvalbumin rich and parvalbumin poor compartments (Kiss et al., 1990; Hontanilla et al., 1998; Henderson et al., 2004; Risold, 2004; Croizier et al., 2010; Mallet et al., 2012). By contrast, the whole substantia innominata contains intermixed neuron populations (Lagos et al., 2012; Lu et al., 2013). MCH axons clearly targeted the cholinergic-rich/parvalbumin-poor regions of the medial septal nucleus, nucleus of the diagonal band, and of the globus pallidus, but they are diffuse within the substantia innominata. MCH and cholinergic neurons have been reported to interact in the medial septal complex and to be involved in sleep control and hippocampal functions such as memory formation (Chung et al., 2011; Lagos et al., 2012; Jego et al., 2013; Lu et al., 2013). However, MCH axons may as well-interact with cholinergic neurons in the substantia innominata and globus pallidus. The cholinergic rich basal telencephalon contains other neuronal types such as GABAergic and glutamatergic neurons that are far more abundant than cholinergic cells (Risold, 2004; Gritti et al., 2006), but little attention has been devoted to date to the innervation that MCH axons may provide to these cells. We observed buttons, and putative synaptic contacts targeting ChAT negative and therefore non-cholinergic cells. This is a topic that will clearly need to be investigated in the future to understand the role of the MCH projections into these regions.

Depending on the location of the injection sites, the numbers, and distributions of FG-labeled neurons were markedly different within the hypothalamus. Injection sites within the medial septal complex retrogradely labeled far more neurons in the hypothalamus than injections centered into the globus pallidus. This was expected because the medial forebrain bundle originates in and innervates the septal region and the substantia innominata, while the globus pallidus is associated to the internal capsule and the cerebral peduncle. We also observed a topographical arrangement in the origin of these projections, with neurons projecting to the medial septal complex being more medial than those projecting to the anterior substantia innominata or globus pallidus. Because some of these projections continue farther to reach the pallium, this topographical organization is clearly reminiscent of that described by Saper (1985) after cortical retrograde tracer injections.

Neurons double labeled with MCH and FG display similar distribution patterns in the hypothalamus. There are more MCH neurons projecting into the medial septal complex than into the globus pallidus. However, several points need to be emphasized:

First, although more abundant after the medial septal complex injections, overall these MCH/FG double-labeled neurons were intermixed with many retrogradely labeled non-MCH neurons in the hypothalamus. By contrast, after FG injections into the globus pallidus, most retrogradely labeled neurons in the hypothalamus were MCH positive. This indicates that MCH neurons may have an important and specific role in relaying the results of the hypothalamic (lateral hypothalamic) processing into the striatonigral system.
Second, in none of our experiments ventromedial-most MCH neurons adjacent and in the periventricular nucleus were labeled. These very medial MCH cells may serve different functions than those in the LHA and dorsal hypothalamus. They are suspected to provide an innervation of the arcuate nucleus and are not observed in mice (Croizier et al., 2010). Then, they probably form a specific MCH sub-population in the rat hypothalamus.

### Sparse indirect connections with the dorsal striatum, innervation of the subthalamic nucleus

Neurons that were retrogradely labeled after FG injections centered into the caudoputamen nucleus received overall a very modest MCH innervation. Only the very ventromedial sector of the pars compacta of the substantia nigra received a clear MCH input, but this region might very well be also associated to the ventral striatum (nucleus accumbens) (Gerfen and Wilson, 1996).

Only significant putative contacts concerned nuclei for which the dorsal striatum is a secondary target or that have multiple projection sites including the striatum. For example, the dorsal raphe nucleus contained quite abundant MCH buttons, some of which adjacent to FG positive neurons after striatal injections. However, the dorsal raphe nucleus project in multiple telencephalic sites and its neurons often send collaterals in many nuclei or territories (Waselus et al., 2011). Therefore, the MCH input that we observed in this nucleus cannot be interpreted as a strong evidence of exclusive indirect dorsal striatal control.

We noted an intense MCH innervation in the subthalamic nucleus. Some neurons of this nucleus were retrogradely labeled after striatal injections, but its main projections are for the globus pallidus as well as the pars reticulata of the substantia nigra. MCH projections in the subthalamic nucleus have not been investigated so far. MCH-R1 seems not to be abundantly expressed in the rodent subthalamic nucleus (Saito et al., 2001). However, MCH neurons contains other neuropeptides as CART and Nesfatin, but most importantly, they are GABAergic (Sapin et al., 2010; Del Cid-Pellitero and Jones, 2012). Therefore, they may act as inhibitory GABAergic neurons. These projections into the subthalamic nucleus were noted in other species (Croizier et al., 2010; Chometton et al., 2014), and should deserve an increased attention in the future.

### MCH neurons are important actors in the basal nuclei circuitry through pallidal and subthalamic nucleus projections

The hypothesis that the MCH system is anatomically associated with the extrapyramidal pathway was advanced by Karl Knigge which unfortunately published too few papers on this topic (Knigge et al., 1996). He advocated quite elegantly that these neurons are in good position to influence the mesostriatal pathway. Since, other authors have convincingly shown that MCH acts on parts of the nucleus accumbens in which a high expression of MCH-R1 is reported (Georgescu et al., 2005; Pissios et al., 2008; Guesdon et al., 2009; Sears et al., 2010; Hopf et al., 2013). Interactions between MCH and mesostriatal pathways have also been reported (Chung et al., 2011). In the present study, we observed that MCH influence on the basal nuclei/extrapyramidal circuitry involved mostly pallidal nuclei as well as the subthalamic nucleus in the rat.

**Figure 6:**
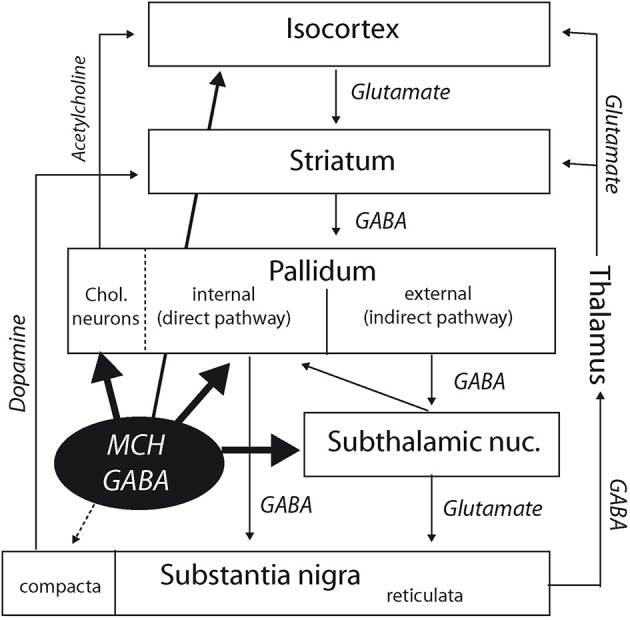
**Diagram summarizing interactions of the MCH systems with the cortico-striato-pallido-nigral (extrapyramidal) pathways**. Besides direct cortical projections, MCH neurons may indirectly influence the cortex by innervating the cholinopetal projections from the internal segment of the globus pallidus. By projecting into this segment of the globus pallidus and in the subthalamic nucleus, they are also in a good position to influence the extrapyramidal direct, and indirect pathways. Finally, the medial section of the pars compacta of the substantia nigra is also targeted by a modest MCH input.

Classically, descending pathways from the striatum take two distinct routes: a direct one that innervates the internal segment of the globus pallidus and the substantia nigra, and an indirect one that involves the external segment of the globus pallidus, subthalamic nucleus, and the substantia nigra (Figure 6). Therefore, MCH axons are susceptible to influence both descending routes from the striatum in the rat: into the internal segment of the globus pallidus for the direct pathway, or by innervating the subthalamic nucleus for the indirect pathway. The role of the subthalamic nucleus within this circuitry has been reevaluated in the last decade. From a motor function, it is now admitted that this nucleus has significant cognitive roles as well (Baunez et al., 2011). It is seen now as a central hub controlling important aspects of voluntary motor output. It is an important target for treatment of Parkinson disease by deep brain stimulation approaches. The role of MCH projections into this nucleus is unclear yet, but considering that MCH neurons are active during REM sleep, these projections into the globus pallidus, and subthalamic nucleus may be important to control motor responses as the cerebral cortex is the siege of an intense electrical activity.

## Author contributions

Conceived and designed the experiments: Pierre-Yves Risold, Dominique Fellmann. Performed the experiments: Sandrine Chometton, Vesna Cvetkovic-Lopes. Analyzed the data: Sandrine Chometton, Vesna Cvetkovic-Lopes, Pierre-Yves Risold, Dominique Fellmann. Contributed reagents/materials/analysis tools: Gabrielle Franchi, Christophe Houdayer, Fabrice Poncet, Amandine Mariot. Wrote the paper: Sandrine Chometton, Pierre-Yves Risold.

### Conflict of interest statement

The authors declare that the research was conducted in the absence of any commercial or financial relationships that could be construed as a potential conflict of interest.

## Abbreviations

AHN: anterior hypothalamic nucleus
AM: anteromedial nucleus thalamus
ARH: arcuate nucleus hypothalamus
BA: bed nucleus accessory olfactory tract
CART: cocaine and amphetamine regulated transcript
ChAT: choline acetyltransferase
Chol: cholinergic
COA: cortical nucleus amygdala
cpd: cerebral peduncle
CP: caudoputamen
da: dopaminergic group in ZI
DGlb: dentate gyrus, lateral blade
DMH: dorsomedial nucleus hypothalamus
FF: fields of Forel
fx: columns of the fornix
GABA: gamma aminobutyric acid
GPe: globus pallidus, external segment
GPi: globus pallidus, internal segment
int: internal capsule
LHA: lateral hypothalamic area
MA: magnocellular preoptic nucleus
ME: median eminence
MEA: medial nucleus amygdala
ml: medial lemniscus
MM: medial mammillary nucleus
MS: medial septal nucleus
mtt: mammillothalamic tract
NDB: nucleus of the diagonal band
opt: optic tract
Parv: parvalbumin
PH: posterior hypothalamic nucleus
PM: premammillary nucleus
PMv: ventral premammillary nucleus
pm: principal mammillary tract
PR: perireuniens nucleus
PV: periventricular nucleus hypothalamus
PVH: paraventricular nucleus hypothalamus
RCH: retrochiasmatic area
REM: rapid eye movement
RE: nucleus reuniens
SBPV: subparaventricular zone hypothalamus
SF: semptofimbrial nucleus
SIc: substantia innominata, caudal part
SIr: substantia innominata, rostral part
SMT: submedial nucleus thalamus
SN: substantia nigra
SNc: substantia nigra, compact part
SNr: substantia nigra, reticular part
SO: supraoptic nucleus
SPF: subparafascicular nucleus thalamus
STN: subthalamic nucleus
SUMl: supramammillary nucleus, lateral part
sup: supraoptic commissures
TM: tuberomammillary nucleus
V3: third ventricle
VM: ventral medial nucleus thalamus
VMH: ventromedial nucleus hypothalamus
VTA: ventral tegmental area
ZI: zona incerta
